# Hypoxic repression of pyruvate dehydrogenase activity is necessary for metabolic reprogramming and growth of model tumours

**DOI:** 10.1038/srep31146

**Published:** 2016-08-08

**Authors:** Tereza Golias, Ioanna Papandreou, Ramon Sun, Bhavna Kumar, Nicole V. Brown, Benjamin J. Swanson, Reetesh Pai, Diego Jaitin, Quynh-Thu Le, Theodoros N. Teknos, Nicholas C. Denko

**Affiliations:** 1 Institute of Virology, Biomedical Research Center, Slovak Academy of Sciences, Bratislava, 84505, Slovak Republic; 2 Department of Radiation Oncology, Ohio State University Comprehensive Cancer Center and Wexner Medical Center, Columbus OH 43210, USA; 3 Department of Otolaryngology, Ohio State University Comprehensive Cancer Center and Wexner Medical Center, Columbus OH 43210, USA; 4 Department of Biomedical Informatics, Center for Biostatistics, Ohio State University Comprehensive Cancer Center and Wexner Medical Center, Columbus OH 43210, USA; 5 Department of Pathology, Ohio State University Comprehensive Cancer Center and Wexner Medical Center, Columbus OH 43210, USA; 6 Department of Pathology, University of Pittsburg, Pittsburg PA 15213, USA; 7 Department of Immunology, Weizmann Institute of Science, Rehovot, Israel; 8 Department of Radiation Oncology, Stanford University School of Medicine, Stanford CA 94305, USA.

## Abstract

Tumour cells fulfil the bioenergetic and biosynthetic needs of proliferation using the available environmental metabolites. Metabolic adaptation to hypoxia causes decreased mitochondrial function and increased lactate production. This work examines the biological importance of the hypoxia-inducible inhibitory phosphorylations on the pyruvate dehydrogenase E1α subunit. Pancreatic cancer cell lines were genetically manipulated to alter the net phosphorylation of PDH E1α through reduced kinase expression or enhanced phosphatase expression. The modified cells were tested for hypoxic changes in phosphorylated E1α, mitochondrial metabolism and growth as xenografted tumours. Even though there are four PDHK genes, PDHK1 is essential for inhibitory PDH phosphorylation of E1α at serine 232, is partially responsible for modification of serines 293 and 300, and these phosphorylations are necessary for model tumour growth. In order to determine the clinical relevance, a cohort of head and neck cancer patient biopsies was examined for phosphorylated E1α and expression of PDHK1. Patients with detectable 232 phosphorylation or expression of PDHK1 tend to have worse clinical outcome. These data show that PDHK1 activity is unique and non-redundant in the family of PHDK enzymes and a PDHK1 specific inhibitor would therefore have anti-cancer activity with reduced chance of side effects from inhibition of other PDHKs.

Tumour growth is limited by the amount, and efficient use of the metabolites that are available for essential biosynthetic and bioenergetic processes^1^. Tumour cells adapt their metabolism to this limitation by shifting the balance of energy production away from oxidative metabolism to a more glycolytic source. It is not entirely clear how the increased reliance on glycolysis promotes tumour growth, but it is thought to increase available precursors for biosynthetic processes^1^. Even less well understood is how the downregulation of mitochondrial function is beneficial. However, clinical data from FDG-PET scans support the concept that tumours are highly glucose avid^2^.

In addition to aberrant oncogenic signals, tumour cells are subject to unique microenvironmental conditions that can impact cellular energetics, such as hypoxia. Extensive literature describes the metabolic response of tumour cells to hypoxia^3^. The induction of the HIF-1 transcription factor increases expression of many genes that stimulate glycolysis, contributing to high efficiency glycolytic conversion of glucose, 2 ADP and 2 NAD^+^ to 2 pyruvate, 2 ATP and 2 NADH^4^. The fate of pyruvate can follow several paths, most commonly mitochondrial oxidation or cytoplasmic conversion to lactate to regenerate NAD^+^ for subsequent glycolysis. Warburg described how tumour cells also have decreased oxidative function, and as a result produce increased amounts of acidic lactate, even when oxygen is present^5^.

One mechanism by which tumour cells have reduced mitochondrial oxidation is presumed to be through hypoxic reduction in pyruvate dehydrogenase (PDH) activity^6^,^7^. There are four pyruvate dehydrogenase kinases in humans that can phosphorylate one of three inhibitory serine residues on the E1α component of pyruvate dehydrogenase^8^. In addition, there are two phosphatases that remove these inhibitory phosphates and re-activate PDH. We, and others, have previously shown HIF-1 dependent induction of the expression of the pyruvate dehydrogenase kinase 1 and 3 genes^6^,^7^,^9^. *In vitro* work on purified proteins has shown that PDHK1 is unique in its ability to phosphorylate the E1α serine at 232, however *in vivo* data has not been reported. This suggests a unique relationship between hypoxia, PDHK1, pSer232-E1α, and regulation of mitochondrial function. Here we generate functional data that show how pancreatic cancer cells induce PDHK1 in hypoxia, increase phosphorylation at all three phosphorylation sites, reduce PDH activity, and reduce mitochondrial oxygen consumption. We also show that this hypoxic circuit is necessary for the metabolic reprogramming of cancer cells for growth *in vivo*, even in the presence of the other three PDHKs. We show for the first time *in vivo* that phosphorylation of serine 232 is absolutely dependent on PDHK1, and the expression of PDHK1 and presence of phosphorylated serine 232 on E1α shows trends of poor outcome in patients with head and neck squamous cell carcinoma.

## Results

### HIF stabilization reprograms cancer cell metabolism to a more Warburg-like phenotype

**Figure 1:**
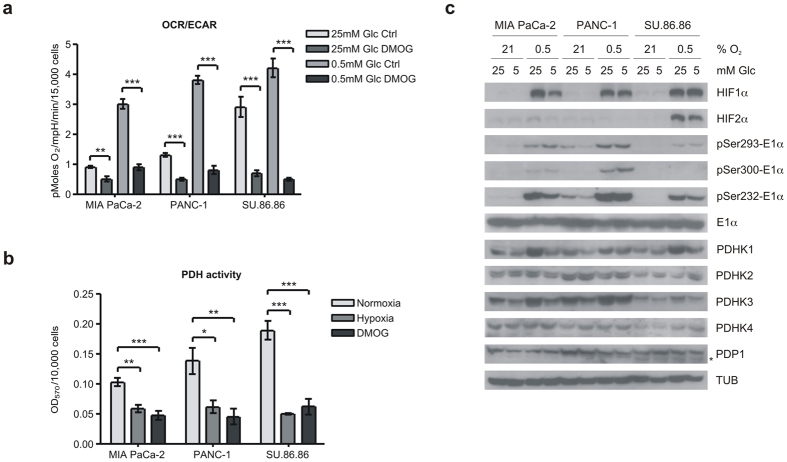
Hypoxia inhibits mitochondrial OCR and PDH activity and induces PDHK1 protein and activity. (**a**) Ratio of oxygen consumption rate (OCR) to extracellular acidification rate (ECAR) measured by Seahorse XF in MIA PaCa-2, PANC-1, and SU.86.86 cell lines in high (25 mM) or low (0.5 mM) glucose incubated overnight with or without 1 mM DMOG. (mean ± SEM, two-tailed Student’s t-test, **p < 0.01, ***p < 0.001) (**b**) Cell-based PDH activity assay in cells incubated 16 h in normoxia, hypoxia (0.5% O_2_) or 1 mM DMOG. (mean ± SD, one-way ANOVA, *p < 0.05, **p < 0.01, ***p < 0.001) (**c**) Western blots of HIFα isoforms, pyruvate dehydrogenase kinase isoforms (PDHKs), phosphatase (PDP1 – lower band *), target phosphorylated serine residues on E1α and total E1α after overnight incubation in normoxia or hypoxia (0.5% O_2_) at 25 or 5 mM glucose as indicated.

We treated three pancreatic cancer cell lines with the prolyl hydroxylase inhibitor DMOG to stabilize HIFα proteins, and measured the resulting metabolic changes using the Seahorse XF flux analyzer. As we and others have reported, HIF stabilization causes a dramatic decrease in mitochondrial oxygen consumption (OCR)^6^,^7^,^10^,^11^, and a modest increase in extracellular acidification (ECAR). This combination results in a profound change in the ratio of OCR/ECAR (Fig. 1a and Supplementary Fig. S1). ECAR is an indirect measure of lactate production from glycolysis, and 80–90% of ECAR can be eliminated by the addition of 2-deoxyglucose (data not shown). We determined the effect of HIF stabilization in both the super-physiological glucose levels normally used in cell culture (25 mM), as well as low glucose (0.5 mM). We find that high glucose levels enhance the glucose consumption of the glycolytic tumour cells (i.e. Crabtree effect). Therefore, in high glucose, there is a significant decrease in OCR, but no change in the glycolytic ECAR of the DMOG-treated cells (Supplementary Fig. S1). In low glucose there is also the decrease in OCR, but in these conditions we detect a significant increase in ECAR (Supplementary Fig. S1). The Seahorse experiments were not performed on hypoxic cultures because of technical limitations of the machine, so we decided to directly measure the activity of pyruvate dehydrogenase enzyme complex with a biochemical assay that measures pyruvate-dependent NADH production. This assay, which was performed on permeabilized cells in the 96-well plate format, shows a significant (40–70%) drop in PDH activity in cells treated overnight with either DMOG or 0.5% oxygen (Fig. 1b).

We next determined the molecular events involved in reduced PDH activity. We determined expression level of the inhibitory kinases (PDHKs) and stimulatory phosphatase (PDP1) in these cell lines incubated in normoxia or hypoxia in high (tissue culture 25 mM) glucose or physiological (5 mM) glucose (Fig. 1c). As reported previously, PDHK1 and PDHK3 expression were both induced by HIF activation^6^,^7^,^9^ in all three cell lines, whereas PDHK2, PDHK4, and PDP1 levels were unaffected (Fig. 1c). The net result of these expression changes can be determined by quantitation of the phosphorylation levels of the target serine residues on the PDH E1α subunit. Using antibodies specific for the phospho modification, we probed the same lysates for total E1α protein and phospho serines 232, 293 and 300. As can be seen in Fig. 1c, all three sites showed significant phosphorylation in response to hypoxia. Serine 232 appeared most robust, and this site has been reported to be phosphorylated only by PDHK1^12^. Similar changes in protein expression were seen in cells treated with DMOG (Supplementary Fig. S2), although the magnitude of the phospho changes may be less.

### Hypoxic phosphorylation of PDH E1α serine 232 requires PDHK1

**Figure 2:**
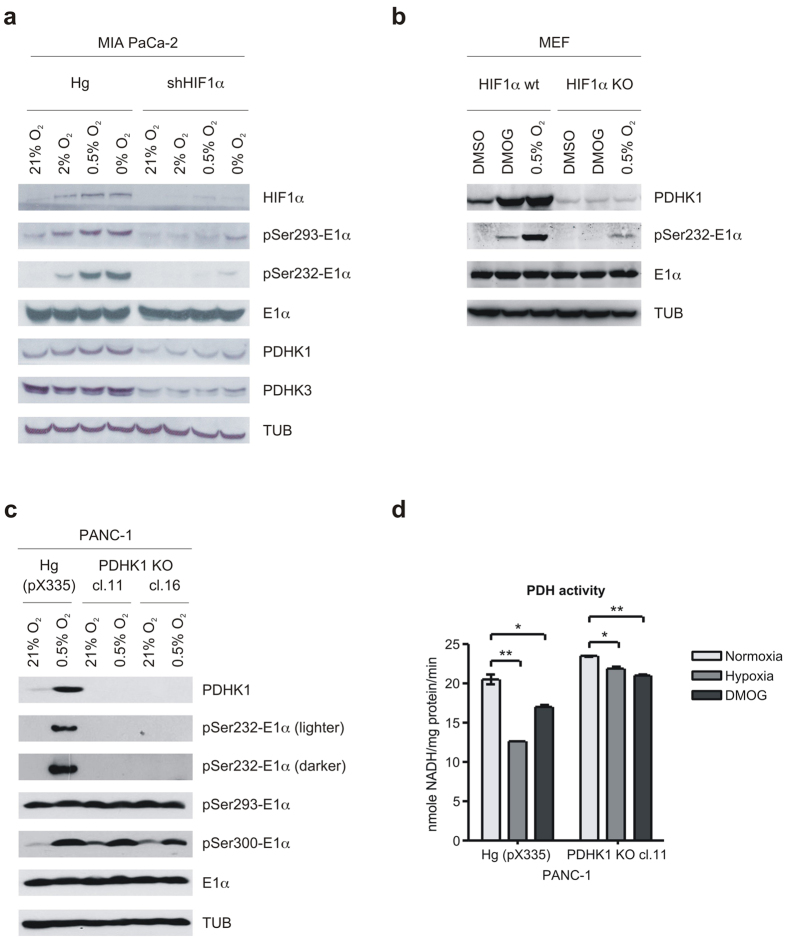
PDHK1 kinase activity can be induced by hypoxia, and it is essential for serine 232 phosphorylation. (**a**) Western blots of MIA PaCa-2 control (Hg) and shHIF1α cells treated for 16 h with decreasing oxygen levels showing effect on HIF-regulated PDHK1 and 3 expression and phosphorylation of PDH E1α. (**b**) WT and HIF1α KO MEFs were treated with hypoxia (0.5% O_2_) or 1 mM DMOG and levels of PDHK1 and pSer232-E1α detected by Western blotting. (**c**) PANC-1 control (Hg (pX335)) and PDHK1 null cells (PDHK1 KO, two clones) treated with hypoxia (0.5% O_2_) overnight showing no kinase activity on Ser232 of E1α. Note that phosphorylation of Ser293 and 300 remain unaffected, reflecting activity of the remaining PDHKs. (**d**) PDH activity measured in extracts of PANC-1 control and PDHK1 null cells described in (**c**), after treatment with hypoxia (0.5%) or 1 mM DMOG overnight. (mean ± SD, one-way ANOVA, *p < 0.05, **p < 0.01).

Hypoxia increases PDHK1 expression and E1α phosphorylation at least in part through a HIF1-dependent mechanism. We decided to investigate this connection with stable knockdown of HIF1α in MIA PaCa-2 cells using reported techniques^13^. Stable shRNAHIF1α resulted in almost complete loss of HIF1α protein (Fig. 2a). We therefore exposed the control and knockdown cells to either 21, 2, 0.5 or 0.01% oxygen and examined PDHK1 and PDHK3 protein expression and E1α phosphorylation. PDHK1 and PDHK3 were induced modestly in the control cells, and the basal levels are reduced and induction eliminated in the knockdown cells (Fig. 2a). Examination of phospho-E1α sites in control cells shows a different pattern of induction by reduced oxygen. Phosphorylated Ser232 and Ser293 increase in a graded manner as oxygen decreased, in contrast, the level of PDHK1 expression rises to a maximum at 2% oxygen and remains constant as oxygen is further reduced (Fig. 2a). At very low oxygen tension, there also appears to be detectable phosphorylation of serine 232 and 293 in the HIF knockdown cells, suggesting that the small amount of PDHK1 is highly active, or another PDHK can phosphorylate serine 232. We interpret these results to indicate that there is either increased PDHK1 activity, or decreased PDP activity at very low levels of oxygen.

In order to formally investigate possible changes in PDHK1 activity, we examined the level of PDHK1 protein and E1α phospho serine 232 in fibroblasts derived from either WT or HIF1α knockout embryos^14^. Both cell lines were treated with either DMOG or 0.5% oxygen and levels of PDHK1, total E1α and phospho-serine 232 were determined (Fig. 2b). We see similar robust levels of PDHK1 protein in the WT cells with either treatment, however, the amount of phospho serine 232 is much greater in the hypoxia-treated cells when compared to the DMOG treated. This also suggests that the activity of the hypoxic protein is greater than that of the DMOG protein. Furthermore, in the HIF1α KO cells, there is no protein induction of PDHK1; however, there is detectable phospho serine 232 in the hypoxic culture. These findings support the idea that either the activity of PDHK1 can be regulated independently from the level of the enzyme, or that one of the other PDHKs can phosphorylate serine 232 in hypoxia.

We addressed the possible contribution of other PDHKs for phosphorylation of E1α in hypoxia by making a PDHK1 null line using CRISPR/Cas9 genome editing. PANC-1 cells were transfected with constructs targeting PDHK1 and individual clones isolated. Approximately 20% of clones were null by Western blot for PDHK1 expression and two were tested for hypoxia-dependent 232 phosphorylation. Figure 2c shows no detectable phospho 232 signal by Western blot, establishing that *in vivo* PDHK1 is the only kinase capable of this modification. Note that serine 300 demonstrated increased phosphorylation, indicating that other kinase(s) are still active in these cells. However, the essential role of PDHK1 is seen in Fig. 2d, where PDHK1 null cells are unable to reduce PDH activity in response to either HIF stabilization (DMOG) or hypoxia.

### Genetic inhibition of E1α phosphorylation reverses hypoxic PDH activity and mitochondrial function

**Figure 3:**
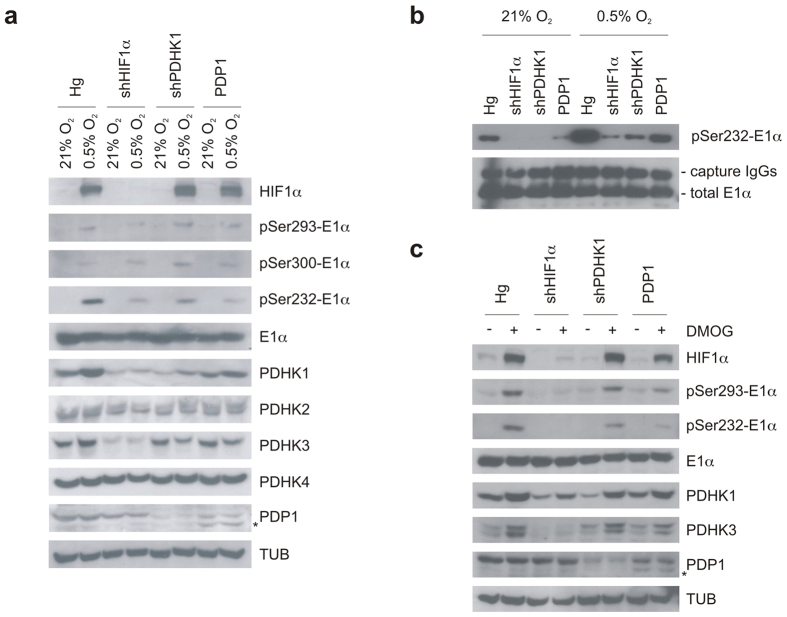
Genetic manipulations can alter PDH E1α phosphorylation in response to hypoxia. (**a**) MIA PaCa-2 control (Hg), silenced HIF1α (shHIF1α) or silenced PDHK1 (shPDHK1) and PDP1 overexpressing cells were incubated for 16 h in normoxia or hypoxia (0.5% O_2_) and phospho-serine E1α detected by Western blot showing that all three modifications can have an inhibitory effect on hypoxic Ser232 phosphorylation. (**b**) Increased pSer232-E1α detection after E1α immunoprecipitation from lysates of cells treated as in (**a**). (**c**) The same analysis as in (**a**) after treatment with 16 h 1 mM DMOG.

To examine the biochemical significance of PDHK1 and pSer232-E1α, we prepared a series of genetically matched MIA PaCa-2 cells that altered this pathway at different levels. We first reproduced the stable HIF1α shRNA knockdown to reduce PDHK1’s hypoxic induction, then directly reduced PDHK1 with specific PDHK1 shRNA knockdown, and finally overexpressed the PDP1 phosphatase. All three of these manipulations should reduce E1α phosphorylation by different mechanisms. We incubated the cells in hypoxia (Fig. 3a,b) or with DMOG (Fig. 3c) overnight and probed the lysates for PDHK1 expression and phospho E1α. We find reduced HIF1α resulted in reduced PDHK1 and PDHK3 levels. We also found reduced PDHK1 in the shPDHK1 cells, and increased PDP1 in the overexpressing cells (Fig. 3a,c). These manipulated cell lines all show reduced phospho serine 232 in response to hypoxia (Fig. 3a). When we immunocaptured PDH and probed for modified E1α, the detection level increased, and the differences in pSer232 were even more definitive (Fig. 3b).

**Figure 4:**
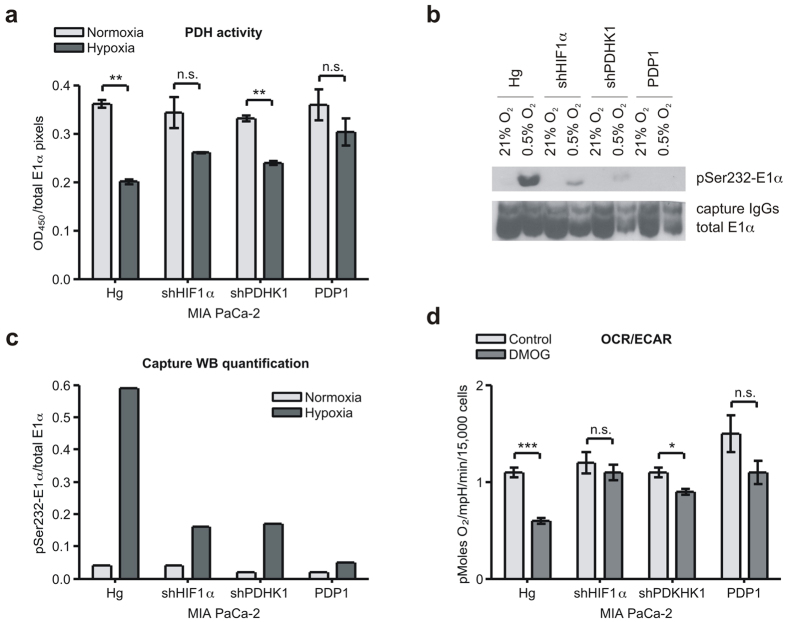
Genetic manipulations can alter hypoxic reduction in PDH activity and OCR. (**a**) PDH activity assay performed on immuno-captured PDH from MIA PaCa-2 Hg, shHIF1α, shPDHK1, and PDP1 cells treated with 16 h normoxia or hypoxia (0.5% O_2_) showing a blunted response to hypoxia. (mean ± SD, two-tailed Student’s t-test, **p < 0.01) (**b**) Western blot of captured PDH from samples in (**a**) showing a commensurate reduction in phosphorylation of E1α. (**c**) Quantization of the phospho signal in panel (**b**) normalized to the signal for total E1α in (**b**) by ImageJ software. (**d**) OCR to ECAR ratios of modified MIA PaCa-2 cells incubated with or without 1 mM DMOG overnight showing modification of hypoxic OCR/ECAR. (mean ± SEM, two-tailed Student’s t-test, *p < 0.05, ***p < 0.001).

In order to directly determine the effect of these manipulations on PDH activity, we used an immune-capture protocol to purify native PDH after either 16 h normoxia or 0.5% oxygen treatment. We used this captured PDH to measure pyruvate-dependent production of NADH, and showed that the genetic manipulations reduced hypoxic downregulation of this captured PDH complex (Fig. 4a). To extend this analysis, we collected the captured PDH from the wells and fractionated it for Western analysis (Fig. 4b,c). Normoxic PDH activity of all the cell types was similar, and the hypoxic samples all showed less decrease in hypoxic PDH activity (Fig. 4a–c).

We next determined mitochondrial response to HIF stabilization by DMOG in these lines (Fig. 4d and Supplementary Fig. S1). We find that all the manipulations resulted in a modest 10–20% increase in baseline normoxic OCR, suggesting low level phosphorylation in normoxic growth (see Fig. 3b). DMOG was unable to enhance the Warburg metabolism (OCR/ECAR) in the shHIF1α and shPDHK1 cells, but had a partial effect on the PDP1 overexpressing cells (Fig. 4d and Supplementary Fig. S1). These results therefore indicate that PDHK1 is a major determinant of the hypoxic shift to a more Warburg metabolism.

### Hypoxic regulation of PDH activity is necessary for model tumour growth

**Figure 5:**
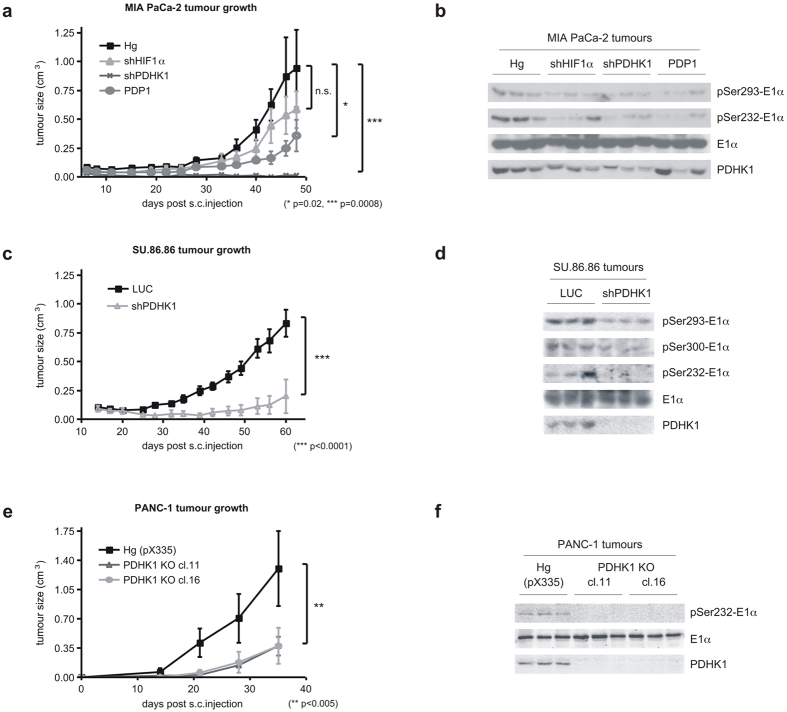
Decreased hypoxic response of PDH activity can slow the growth of xenografted tumours. (**a**) Tumour growth curves of MIA PaCa-2 genetically modified cells (HIF1α and PDHK1 knockdown, PDP1 overexpression) described in Fig. 4 grown in nude mice (n = 8 per group in replicate experiments). Statistically significant differences exist between control (Hg) and shPDHK1 and PDP1 tumours as indicated. (mean ± SEM, two-way ANOVA) (**b**) Western blots from three random tumours of each group in (**a**) showing decreased PDHK1 expression and activity *in vivo*. (**c**) Tumour volumes of SU.86.86 control (LUC) and PDHK1 knockdown cells grown in nude mice (n = 8 per group). The growth rate differences were statistically significant. (mean ± SEM, two-way ANOVA) (**d**) Western blots from three random tumours in (**c**) showing decreased PDHK1 expression and activity *in vivo*. (**e**) Tumour volumes of PANC-1 control (Hg (pX335)) and 2 clones of PDHK1 null cells (PDHK1 KO) described in Fig. 2 grown in nude mice (n = 4 per group). The growth rate differences were statistically significant. (mean ± SEM, two-way ANOVA) **(f)** Western blots from three random tumours in (**e**) showing no PDHK1 expression and activity *in vivo*.

To establish what effect that reduced hypoxic phospho serine 232-E1α would have on the growth of transplanted tumours, we inoculated these manipulated MIA PaCa2 cells into immunocompromised mice. All of these cell lines displayed some reduction in growth as tumours (Fig. 5a). As has been reported, shHIF1α showed a modest reduction in growth^15^, while shPDHK1 showed the most dramatic effect^16^. HIF1α controls the expression of many genes, some of which may have opposite impact on tumour growth than PDHK1. PDP1 overexpression showed intermediate growth inhibition. We harvested these tumours to check for PDHK1 expression and E1α phosphorylation (Fig. 5b). The shPDHK1 and PDP1 overexpressing tumours displayed the greatest reduction in phospho serine 232 *in vivo*, and had the poorest growth as tumours. We confirmed these findings in the SU.86.86 cells and found that PDHK1 knockdown also resulted in a significantly suppressed tumour growth (Fig. 5c), and reduced E1α phosphorylation on serine 232 and 293 (Fig. 5d). Interestingly, when we tried to repeat these experiments in the PANC-1 cells, we were unable to completely knockdown PDHK1. The minimally remaining PDHK1 still fully phosphorylated serine 232-E1α (Supplementary Fig. S2). These PANC-1 shPDHK1 cells formed tumours at a rate indistinguishable from the parental PANC-1 (Supplementary Fig. S2). However, when we completely knocked out PDHK1 in the PANC-1 cells using CRISPR, these cells were completely unable to phosphorylate serine 232 on E1α (Figs 2c and 5f), and grew significantly slower when transplanted into immune-deficient mice (Fig. 5e). These results support the concept that it is the hypoxic ability of PDHK1 to phosphorylate E1α that is necessary for efficient model tumour growth.

We examined the growth of these cells by colony formation assay in hypoxia *in vitro* to determine if we could identify why they would not grow as tumours. This analysis shows that while there is little difference in the growth of the various cells in mild hypoxia (2% oxygen data not shown), there is significant inhibition of the growth of the PDHK1 knockdown cells in the stringent 0.5% oxygen environment (Supplementary Fig. S2).

### PDHK1 and pSer232 can predict for poor outcome in patients with HNSCC

**Figure 6:**
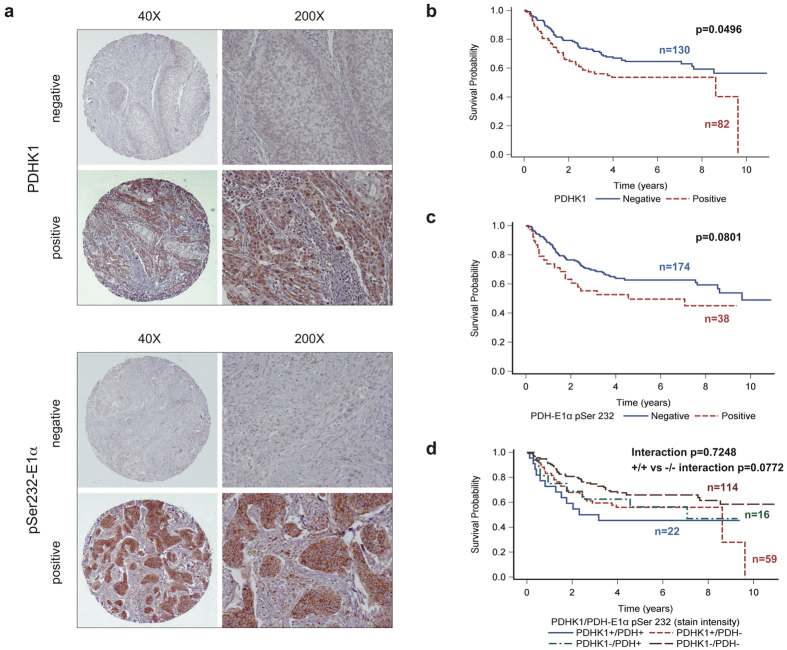
PDHK1 and pSer232-E1α are prognostic markers in oropharyngeal cancers. (**a**) Examples of low (left) and high (right) magnifications images of staining patterns for negative and positive cores detecting either PDHK1 (top) or PDH pSer232-E1α (bottom). (**b**) Kaplan Meier (KM) survival curves showing univariate analysis of overall survival based on PDHK1 stain intensity. (**c**) KM curves showing univariate analysis of survival based on pSer232-E1α stain intensity. (**d**) Dual marker analysis combining both markers.

In order to determine if these findings were pertinent to spontaneous human tumours, we performed histochemical analysis on a tissue microarray (TMA) derived from a total of 223 oropharyngeal tumours treated at OSU James. The TMA and patient characteristics were described previously^17^,^18^. Univariate analyses for PDHK1 stain intensity biomarker showed it was predictive for overall survival at the p = 0.05 level with a hazard ratio of 1.52 (95% CI 1.001, 2.309; p = 0.0496) (Fig. 6b and Supplementary Fig. S3). These results are similar to what has been reported for lung cancers^19^. Univariate analysis of pSer232-E1α stain intensity was marginally predictive for overall survival with Fig. 6c showing the positive tumours predicting a 1.56 fold increased risk of death (95% CI 0.948, 2.567; p = 0.0801).

The effect of dual marker interactions on survival was assessed between PDHK1 and pSer232-E1α stain intensity. Interestingly, this interaction was not statistically significant (p = 0.7248). However, although the interaction of the two markers was not significant, we noticed a trend of those negative for both markers demonstrating better overall survival than those positive for both markers, as evidenced by a substantial difference between the two (p = 0.0772) (Fig. 6d). However, almost half of the cases scored either PDHK1 + /pSer232- or PDHK1-/pSer232+, indicating that factors other than PDHK1 levels dictate the amount of phosphorylation of E1α at serine 232.

## Discussion

It has been known for 50 years that reversible phosphorylation can regulate pyruvate dehydrogenase activity^20^. The identification of the kinases and phosphatases confirmed that they could regulate the pyruvate dehydrogenase complex (PDC) *in vitro*^21^. However, the complexity of the system has made it difficult to determine how this mitochondrial regulatory circuit contributes to a metabolic program *in vivo* that supports tumour growth. More recent work has proposed the concept that regulation of PDHK1 activity by growth factor stimulation^22^,^23^, or hypoxia^16^,^24^, can support tumour growth.

However, these studies did not measure the inhibition of PDC, or determine what exactly was responsible for the pro-tumourigenic effect. Here we show that it is in fact down-regulation of PDC that is necessary, and hypoxia not only regulates the expression of PDHK1, but its kinase activity as well. We also show that PDHK1 is uniquely essential for phosphorylation of serine 232 *in vivo*. Furthermore, we show that those patients with high levels of both PDHK1 and of phospho serine 232 E1α in head and neck cancers tended to have poorer outcome.

Recent reports used metabolic flux analysis to identify processes in tumour cells that regulate mitochondrial function for the production of citrate (for *de novo* synthesis of fatty acids)^25^, and NADPH (also for fatty acid synthesis and buffering of oxidative stress)^26^. For example, DeBerardinis and colleagues identified pyruvate carboxylase as a means for pyruvate to enter the TCA cycle, even with reduced PDC activity^27^. In addition, several groups have shown that the entry of glutamine into the TCA cycle can also contribute to the generation of citrate through reductive carboxylation by isocitrate dehydrogenase^28^,^29^,^30^. The reductive carboxylation of glutamine is favored with HIF1 activation^29^, and this hypoxia-regulated metabolic process is also necessary for the growth of model tumours^25^.

The clinical use of the FDG-PET scan has confirmed that many human tumours are glucose-avid, but it does not indicate how the glucose is utilized within the tumour cell. Hypoxia and HIF1α have been shown to stimulate a Warburg-type of metabolism and be poor prognostic markers of patient outcome in head and neck tumours^31^. Several HIF1 target genes related to glucose metabolism have also been shown to predict poor outcome, such as GLUT1^32^, and even PDHK1^19^. However, our findings suggest that PDHK1 is by far the major driver of altered metabolism when HIF1α is stabilized.

Our data also examine the activity of PDHK1 *in vitro* and *in vivo* by determining phospho serine 232 levels. Interestingly, this marker also suggests a poor patient outcome, but surprisingly does not track with PDHK1 expression (Fig. 6). Patients who are double positive for both markers appear to have the worst outcome, while patients who are double negative appear to have the best outcome.

Recent reports have investigated the importance of PDHK1 in cancer cells, and identified mechanisms for its regulation. Chen and colleagues have shown that receptor tyrosine kinase signalling^23^ can stimulate PDHK1 activity and propose that mitochondrial FGFR can stimulate PDHK1 kinase function. They have also investigated reversible regulation of PDP1 and suggested that tyrosine phosphorylation can also contribute to PDP activity and regulation of PDH^33^. A recent report by the Lu group has identified a site on PDHK1 that can be phosphorylated by phosphoglycerate kinase, and have proposed a mechanism by which hypoxia stimulates mitochondrial translocation of PGK1 to the mitochondria where it phosphorylated PDHK1 and stimulated PDHK1 function^34^.

These results, and that of others, show that PDHK1 activity predicts for poor outcome in head and neck cancer and other clinical sites^35^,^36^,^37^. However, datasets from other studies such as ovarian cancer do not necessarily get the same results^38^. These studies also suggest that PDHK1 kinase activity could be targeted as an anti-cancer strategy. Several groups have tried to inhibit tumour growth using the low activity pan-PDHK inhibitor dichloroacetate^39^. However, it does not appear to be possible to achieve inhibitory concentrations in patients^40^. It has been possible to identify a novel high-affinity inhibitors of PDHKs^41^,^42^. Several inhibitors of PDHK2 have been identified as potential anti-diabetic agents, but they have variable activity on the other PDHK family members. Perhaps a pure PDHK1 inhibitor would be better suited as an anti-cancer agent.

## Materials and Methods

### Cell lines and cell culture

Human pancreatic carcinoma MIA PaCa-2, PANC-1, and SU.86.86 cell lines were purchased and validated by ATCC. These lines were chosen to cover a range of metabolic backgrounds from more glycolytic (PANC-1) to intermediate (MIA PaCa-2) to more oxidative (SU86.86). Wild-type and HIF1α knock-out mouse embryonic fibroblasts (MEF) were a kind gift from Dr. Johnson (Cambridge, UK). Cells were grown in DMEM medium with 10% FBS in high (25 mM), normal (5 mM) or low (0.5 mM) glucose with 0.1% DMSO or 1 mM dimethyloxalylglycine (DMOG, Sigma-Aldrich) as indicated. Hypoxia was achieved in an Invivo_2_ humidified hypoxia workstation (Ruskinn Technologies).

### Western Blotting and Antibodies

Lysates were generated in RIPA buffer containing protease and phosphatase inhibitors (5 mM sodium fluoride, 2 mM β-glycerophosphate, 1 mM sodium orthovanadate, 1 mM phenylmethylsulfonyl fluoride, and Complete Mini protease inhibitor cocktail (Roche)), 20–50 μg of total proteins electrophoresed and blotted to PVDF. Antibodies used were for total PDH E1α (1:6000, MitoSciences), pSer293-E1α (1:1000, EMD Chemicals), pSer300-E1α (1:1000, EMD Chemicals), pSer232-E1α (1:3000, EMD Chemicals), PDHK1 (1:4000, Assay Designs), PDHK2 (1:500, Novus), PDHK3 (1:1000, Novus), PDHK4 (1:1000, Novus), PDP1 (1:1000, Sigma Aldrich), HIF1α (1:1500, BD), HIF2α (1:1000, Novus), tubulin (1:1000, Santa Cruz). Primary antibodies were detected with HRP secondaries (1:4000, Santa Cruz) and visualized with ECL (Amersham) on film or ChemiDoc (Bio-Rad), or with fluorochrome labelled secondary antibodies (Li-Cor) visualized on a Li-Cor Odyssey.

### Plasmids and stable transfections

Stable knockdown (shHIF1α, shPDHK1), overexpressing (PDP1), and knockout (PDHK1 KO) cell lines were created by cotransfection with pTKhygro and pSUPER-shHIF1-α or pSUPER-shPDHK1^6^ or pCMV6-XL5 PDP1 (OriGene) or pX335-crisprPDHK1 A together with pX335-crisprPDHK1 B using Lipofectamine 2000 (Invitrogen), followed by selection in 300 μg/ml hygromycin. Drug-resistant colonies were tested by Western blotting and at least three positive clones were randomly pooled for study. The gRNA/CAS9nickase plasmids were generated by introducing targeting sequences: 5′ CCAGGGTGTGATTGAATACA 3′ for top strand and 5′ TGGGAATGACATCATTGTGT 3′ for bottom strand (pX335-crisprPDHK1 A and pX335-crisprPDHK1 B, respectively) into the BbsI site in pX335-U6-Chimeric_BB-CBh-hSpCas9n(D10A) (Addgene plasmid # 42335).

### PDH activity assays

For whole-cell colorimetric PDH activity assay, 10^4^ cells/well were permeabilized with 0.5% Triton X-100 and incubated in 5 mM 3-bromopyruvate, 1 mM MgCl_2_, 0.05 mM EDTA, 0.0025% Triton X-100, 0.3 mM thiamine diphosphate, 10 μM rotenone, 10 mM pyruvate, 3 mM NAD^+^, 1 mM Co-A, 0.75 mM nitroblue tetrazolium and 0.05 mM phenazine methosulfate in 50 mM Tris-HCl pH 7.8 for 30–60 min at 37 °C. Insoluble blue formazan precipitate was dissolved in 10% SDS in 0.01N HCl overnight. OD at 570 nm is reported.

For immune-captured PDH activity, the PDH Enzyme Activity Microplate Assay (MitoSciences) was used according to the manufacturer. Solubilized cell culture suspension was immunocaptured in 96-well plates using a proprietary PDH capture antibody. The wells were washed, and a reaction buffer was added to measure pyruvate-dependent reduction of NAD^+^to NADH coupled to the reduction of a reporter dye. Absorbance at 450 nm is reported. The wells were then washed in PBS, captured proteins harvested in 1x loading buffer and electrophoresed. Membranes were probed for pSer232-E1α and total E1α. PDH activity was normalized to total E1α.

PDH activity of the PDHK1 KO cells was measured with a colorimetric PDH activity assay kit (Sigma-Aldrich) according to the manufacturer. Cells were homogenized in assay buffer, diluted 10x and absorbance at 450 nm was measured kinetically for approximately 30 min at 37 °C after addition of PDH developer and PDH substrate. Sample blank values (without PDH substrate) were subtracted from the sample readings and PDH activity (nmole NADH/min) normalized to mg protein.

### XF96 Extracellular Flux Analyzer measurements

0.75–1.5 × 10^4^ cells per well (Seahorse XF plate) were treated overnight in regular high (25 mM) or low (0.5 mM) glucose DMEM with or without 1 mM DMOG. Prior to XF assay, medium was changed to high or low glucose bicarbonate-free medium with an adjusted pH to 7.4. Basal oxygen consumption rates (OCR) and extracellular acidification rates (ECAR) were measured three times in quadruplicates and normalized to 15,000 cells. Error bars represent the standard error of the mean.

### Tumour Growth Delay

5 × 10^6^ cells were injected *s.c.* into the flanks of 6-week old female nude mice (four-eight tumours per cell type, in duplicate experiments), and caliper measurements of two perpendicular diameters were used to monitor tumour volumes (volume = (d_1_) × (d_2_)^2^ × 0.52). Three random tumours per cell type were harvested in RIPA and analyzed by Western blotting as described above. Error bars represent the standard error of the mean. All animal experiments were performed following protocols approved by institutional IACUC review, with daily veterinarian oversight.

### Tissue Microarrays

Tissue microarrays (TMA) were constructed from pathology specimens of surgically resected oropharyngeal squamous cell carcinomas removed at the James Cancer Hospital from 2002 to 2009^17^,^18^. Each block was cored 4 times, three cores were taken from the tumour and one from the adjacent normal tissue. The use of archival patient samples was HIPAA compliant and approved by The Ohio State University IRB.

The expression of PDHK1 and pSer232-E1α PDH was assessed by standard immunohistochemistry. Slides were deparaffinized and rehydrated, prior to heat-induced antigen retrieval using the Target Retrieval Solution (Dako) in a decloaking chamber at 120 °C for 20 minutes. Endogenous peroxidase and alkaline phosphatase activity was quenched with the Dual Endogenous Enzyme Block (Dako). The Vectastain Elite ABC Kit (Vector Laboratories) was then used to detect PDHK1 (Enzo Life Sciences) and pSer232-E1α (EMD Biosciences) immunostaining. The expression levels of these proteins were visualized using 3, 3′-diaminobenzidine and slides were counterstained in hematoxylin.

The expression of the proteins was scored blind by a pathologist (BJS). The proportion of tumour cells stained from 0 to 100%, and stain intensity was given a score of 0 (none), 1 (low), 2 (moderate), 3 (high). A quick score was calculated by multiplying the stain proportion and intensity. The scores for the replicate cores were averaged together.

For the purpose of analysis, cut-offs for determining positive PDHK1 and pSer232-E1α status were based on distribution of the expression. PDH pSer232-E1α positive status was defined as: stain intensity average ≥2; stain proportion average ≥50%; and quick score average >20 (median split). For PDHK1, approximately 60% of the patients did not express any immunoreactivity, so PDHK1 positive is stain intensity >0; stain proportion >0%; and quick score >0.

Overall survival was defined as time from the date of surgery, with living patients censored at last observation. Survival curves were plotted using the Kaplan-Meier method. Cox proportional hazards models were used to assess univariate associations of biomarkers as predictors for death. Unadjusted hazard ratios (HR) and 95% confidence intervals (CI) are reported. Multivariable models were explored to assess dual-marker interactions between pSer232-E1α and PDHK1. All analyses were conducted in SAS, version 9.2 (SAS Institute, Cary, North Carolina).

### Statistical analyses

The data are presented as mean ± standard deviation (SD) or standard error of the mean (SEM) where indicated. For comparison between two groups, they were tested by two-tailed Student’s t-test, by one-way ANOVA for multiple comparisons, and by two-way ANOVA for tumour growth curves. P < 0.05 was considered significant (*p < 0.05, **p < 0.01, ***p < 0.001, n.s. non-significant). The analyses were performed using Prism, version 4.0a (GraphPad, La Jolla, CA).

Tumour microarray statistical analyses were described above.

## Additional Information

**How to cite this article**: Golias, T. *et al.* Hypoxic repression of pyruvate dehydrogenase activity is necessary for metabolic reprogramming and growth of model tumours. *Sci. Rep.* **6**, 31146; doi: 10.1038/srep31146 (2016).

## Supplementary Material

Supplementary Information
