# Supplementary material for: Hypoxic repression of pyruvate dehydrogenase activity is necessary for metabolic reprogramming and growth of model tumours
Source: Sci Rep. 2016 Aug 8;6:31146. doi: 10.1038/srep31146 (PMC4976358; doi:10.1038/srep31146)
Supplement: Supplementary Information [file srep31146-s1.pdf]

Hypoxic repression of pyruvate dehydrogenase activity is necessary for metabolic reprogramming and growth of model tumours

Tereza Golias, Ioanna Papandreou, Ramon Sun, Bhavna Kumar, Nicole V Brown, Benjamin J Swanson, Reetesh Pai, Diego Jaitin, Quynh-Thu Le, Theodoros N Teknos, and Nicholas C Denko*

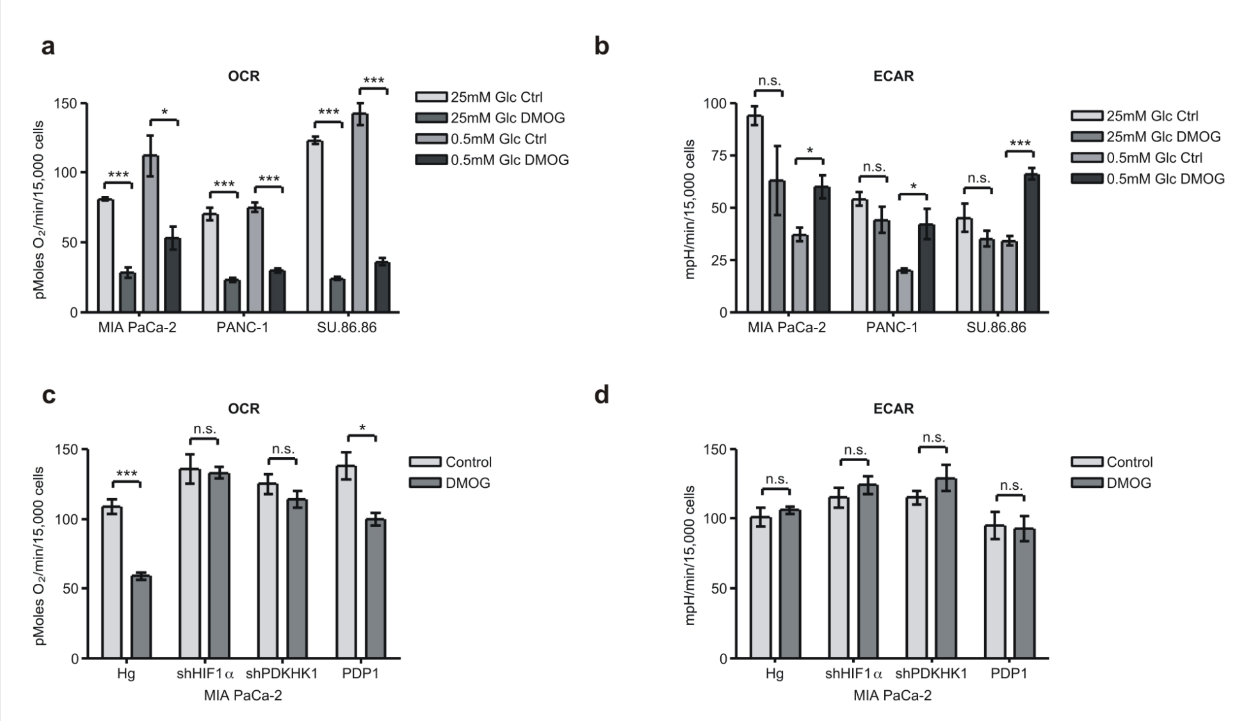

Supplementary Figure S1 (a) Oxygen consumption rates (OCR) measured by Seahorse XF in MIA PaCa-2, PANC-1, and SU.86.86 cell lines in high (25mM) or low (0.5mM) glucose incubated overnight with or without 1mM DMOG. **(b)** Extracellular acidification rates (ECAR) of the same samples as in (a). **(c)** Oxygen consumption rates of modified MIA PaCa-2 cells incubated with or without 1 mM DMOG overnight showing modification of hypoxic OCR. **(d)** Extracellular acidification rates of the same samples as in (c). (All data presented as mean ± SEM, two-tailed Student's t-test, * $p < 0.05$, *** $p < 0.001$)

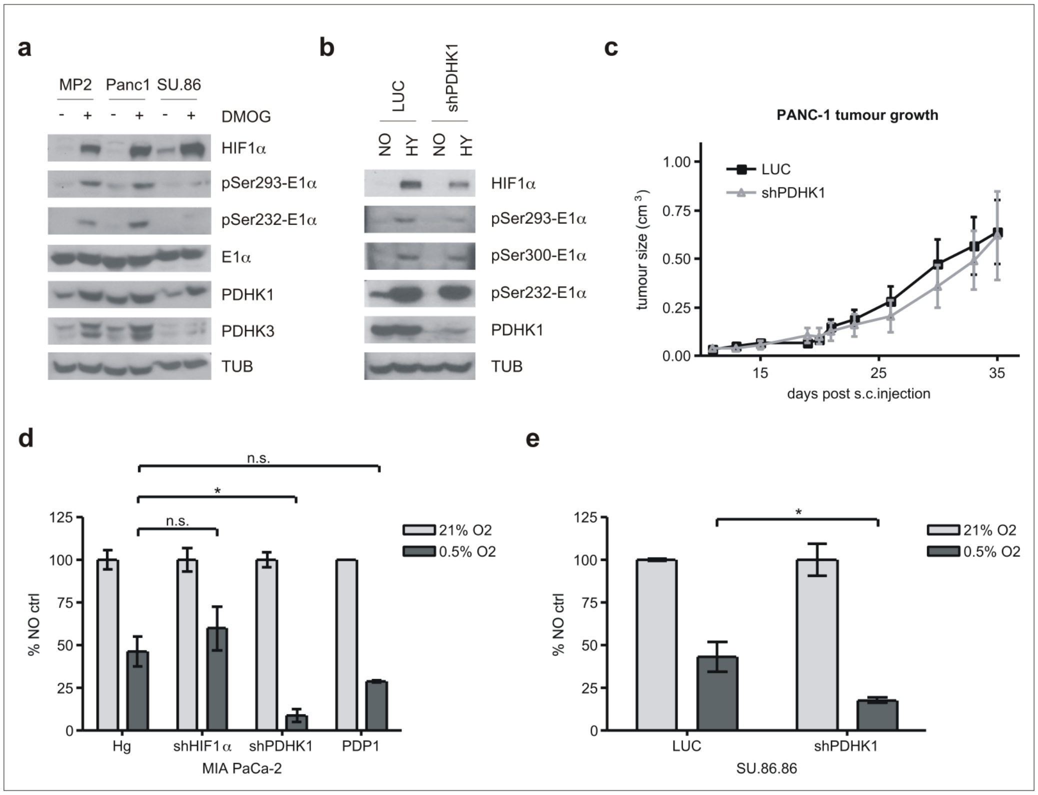

Supplementary Figure S2 (a) Western blots of hypoxia-regulated PDHK1 and PDHK3, and phosphorylated inhibitory serine residues on E1α of PDH in pancreatic cancer cell lines incubated with 0.1% DMSO or 1mM DMOG overnight. **(b)** Western blots of PANC-1 control (LUC) and PDHK1 knocked-down (shPDHK1) cells incubated at 21% or 0.5% O_2 overnight showing that remaining PDHK1 in the silenced cells is still fully able to phosphorylate PDH E1α. **(c)** When PANC-1 cells from (b) were used to grow xenografts, the growth curves show no difference between control and PDHK1 knock-downs supporting the notion that it is PDHK1 regulation of PDH activity that is necessary for tumour growth. **(d)** Colony formation assay of genetically-modified MIA PaCa-2 cells plated at 300 cells per 6cm tissue-culture dish in high glucose (25mM) DMEM and grown in normoxia or hypoxia (0.5% O_2) for two weeks. Colonies were stained with 0.25% crystal violet in ethanol and counted. (mean ± SD, one-way

ANOVA, * $p < 0.05$) **(e)** Colony formation assay of genetically-modified SU.86.86 cells plated and grown under the same conditions as (d). (mean ± SD, two-tailed Student's t-test, * $p < 0.05$) (d) and (e) showing that PDHK1 is advantageous for cell growth under hypoxic conditions.

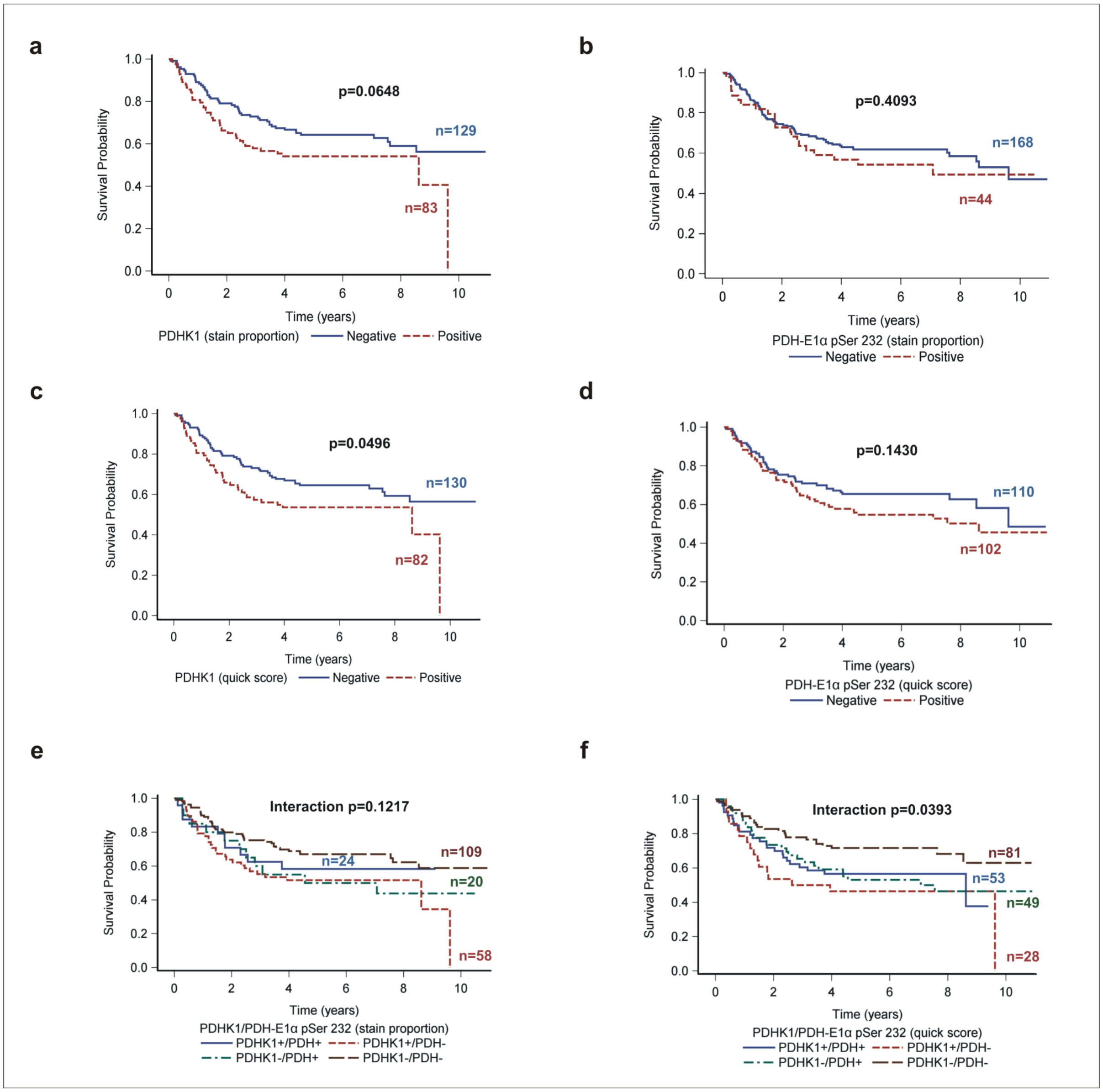

Supplementary Figure S3 Univariate Kaplan Meier survival curves and significance of difference based on scored parameters of the TMA. **(a)** PDHK1 stain proportion **(b)** pSer232-E1α stain proportion **(c)** PDHK1 quick score **(d)** pSer232-E1α quick score. Dual parameter analyses for **(e)** stain proportion and **(f)** quick score.
